# Is ^18^F-FDG PET/CT useful for diagnosing relapsing polychondritis with airway involvement and monitoring response to steroid-based therapy?

**DOI:** 10.1186/s13075-019-2083-8

**Published:** 2019-12-12

**Authors:** Yunxiang Zeng, Minfang Li, Sheng Chen, Lin Lin, Shiyue Li, Jianxing He, Jinlin Wang

**Affiliations:** 1 The State Key Laboratory of Respiratory Disease, China Clinical Research Centre for Respiratory Disease, Guangzhou Institute of Respiratory Health, First Affiliated Hospital of Guangzhou Medical University, 151 Yanjiang Road, Guangzhou, 510120 Guangdong Province China; 2 The Second School of Clinical Medical Sciences, Guangzhou University of Chinese Medicine, Guangzhou, China; 3 Department of Respiratory Medicine, Shenzhen Traditional Chinese Medicine Hospital, Shenzhen, China; 4 Department of Respiratory Medicine, Guangdong Hospital of Traditional Chinese Medicine, Second Clinical Hospital of Guangzhou University of Chinese Medicine, Guangzhou, China

**Keywords:** ^18^F-FDG PET/CT, Relapsing polychondritis, Cartilage, Airway involvement, Corticosteroid, Response to therapy

## Abstract

**Background:**

^18^F-fluorodeoxyglucose (FDG) positron emission tomography/computed tomography (PET/CT) is a promising tool for diagnosing relapsing polychondritis (RP). However, its usefulness in assessing RP with airway involvement is unknown.

**Objective:**

This study aimed to further evaluate and confirm the potency of ^18^F-FDG PET/CT in diagnosing RP with airway involvement and monitoring response to steroid-based therapy.

**Methods:**

A total of 30 patients from a dedicated respiratory centre, diagnosed with RP in accordance with McAdam, Damiani or Levine criteria, were included in this study. All patients underwent baseline ^18^F-FDG PET/CT, and 10 patients underwent second scans after 2.5–15 months of steroid-based therapy. Visual scores (VS) and maximal standard uptake values (SUV_max_) were analysed.

**Results:**

In the initial scan, 83.3% (25/30) of patients were found to have FDG uptake in more than one cartilage. The median VS and SUV_max_ in the cartilages were 3 (range, 1–3) and 3.8 (range, 1.9–17.9), respectively. Positive rates for PET/CT-guided biopsy in nasal, auricular, and tracheal/bronchial cartilages were 100% (5/5), 88.9% (8/9), and 10.5% (2/19), respectively, but the positive biopsy rate in the auricular cartilage was 92.3% (12/13) even without PET/CT assessment. Based on biopsy-proven sites, the sensitivity of PET/CT was 55.6%, and the specificity was 5.3%. Compared with the baseline scan, the second scan showed much lower median VS (2 vs 3, respectively; *p* < 0.0001) and SUV_max_ (2.9 vs 3.8, respectively; *p* < 0.001). Of 10 patients who underwent second PET/CT, 8 had complete therapeutic response, while 2 had partial response.

**Conclusion:**

^18^F-FDG PET/CT assists in identifying multiple cartilage involvement in RP, but it seems neither a sensitive nor specific modality in diagnosing RP with airway involvement. Moreover, PET/CT has limited utility in locating biopsy sites and monitoring therapeutic response to corticosteroids.

## Background

Relapsing polychondritis (RP), first described by Jaksch-Wartenhorst in 1923 [1], is a rare autoimmune inflammatory disease characterised by episodic inflammation of the cartilaginous tissue and proteoglycan-rich structures throughout the body [2, 3]. Currently, the diagnosis of RP is still mainly based on clinical manifestations [4–6]. However, the initial symptoms are often atypical, and the prognosis is unfavourable when the respiratory tract is involved. Airway involvement is a major cause of morbidity and mortality in patients with RP [7] and occurs in up to 50% of patients during the course of the illness [4, 5, 7, 8], but half of these patients had no airway abnormalities on routine inspiratory CT.

In 2007, positron emission tomography/computed tomography (PET/CT) with ^18^F-fluorodeoxyglucose (FDG), as a tracer providing metabolic information on the entire body, was used in a patient with RP [9]. Since then, a few case reports or small case series have demonstrated that PET/CT is capable of determining organ involvement and evaluating disease activity and therapeutic response [10–19]. In 2014, we retrospectively investigated 6 patients with RP and found that PET/CT is a valuable tool in diagnosing RP and monitoring treatment response [20]. Concurrently, Yamashita [21] and Lei [22] analysed a series of patients with RP who undergone PET/CT and concluded that PET/CT is a potentially useful tool for the early diagnosis of RP, especially in patients without easily biopsied organ involvement or with atypical RP. It is also suggested that PET/CT is a radiological tool in the selection of biopsy sites [20–23]. A combination of PET/CT and transbronchial needle aspiration (TBNA) in tracheal/bronchial cartilages was an effective approach of disease diagnosis [22]. However, the value of these investigations was limited by relatively heterogeneous populations and small sample sizes.

In the present study, we retrospectively analysed the data of 30 patients with RP in a respiratory centre. To the best of our knowledge, this is a cohort study with the largest sample size. We aimed to further confirm the value of ^18^F-FDG PET/CT in disease diagnosis. Moreover, we aimed to investigate the potential usefulness of ^18^F-FDG PET/CT in response assessment.

## Patients and methods

### Patients

Patients with RP who visited China Clinical Research Centre for Respiratory Disease, Guangzhou Institute of Respiratory Health, First Affiliated Hospital of Guangzhou Medical University, due to predominant respiratory symptoms between January 2010 and January 2018 were included in the study. The inclusion criteria were adult patients (≥ 18 years) diagnosed with to have RP based on either McAdam’s, Damiani and Levine’s or Michet’s criteria [4–6]. A total of 30 patients met the criteria. Patient nos. 1 and 3 were previously reported in separate case reports [13, 23], and information on 6 patients (nos. 1–6) was retrospectively reviewed [20]. The variables and clinical data of these patients were collected for analysis. This study was approved by the Institutional Review Board of the First Affiliated Hospital of Guangzhou Medical University.

### ^18^F-FDG PET/CT

As described in our previous study [20], patient preparation for ^18^F-FDG PET/CT included the following steps: (1) avoidance of strenuous work or exercise in the preceding 24 h, fasting for > 6 h before the scan, and measurement of fasting blood glucose levels before the scan (which were required to be < 130 mg/dL [7.2 mmol/L] before ^18^F-FDG injection); (2) intravenous injection of ^18^F-FDG in a dosage of 3.70–5.55 MBq/kg together with 10 mg furosemide to accelerate renal ^18^F-FDG elimination; (3) CT scan from the brain to the pelvis using a multidetector spiral CT scanner (3.75-mm slice thickness, pitch 0.875, rotation speed 140 keV, 120 mAs) before the PET scan; and (4) acquisition of whole-body PET images using a PET/CT system (GE Discovery ST; GE Medical Systems Inc., WI, USA) 60 min after tracer administration (acquisition time, 2.5 min per bed position for 6 to 7 bed positions). All the PET-scan in our study is full-body PET-scan, and nearly all the cartilaginous zones can be assessed in our patients.

### Cartilage biopsy

#### Auricular cartilage biopsy

Based on the PET/CT findings, auricular cartilages with FDG accumulation were selected. If there is no FDG uptake, the anterior cartilage was used as the biopsy site. After routine disinfection and local anaesthesia, the auricular cartilage with a diameter of approximately 3 mm was obtained and fixed in formalin. Gauze was placed on the biopsy site.

#### Nasal cartilage biopsy

Biopsies were performed in 5 patients with FDG accumulation in the nasal cartilage by PET/CT. Nasal cartilage biopsies were performed under nasal endoscopy. After routine anaesthesia, the nasal cartilage with a diameter of approximately 3 mm was obtained. If there was no obvious or small amount of bleeding, gauze was placed on the nasal cavity to stop the bleeding. If necessary, bilateral nasal cavities were filled with gauze to stop the bleeding.

#### Tracheal cartilage biopsy by bronchoscopy

Based on the PET/CT findings, patients underwent conventional bronchoscopy with midazolam, sufentanil and lidocaine. The trachea or bronchus with accumulated FDG was biopsied using forceps, and 4–5 tissues were obtained for pathological examination.

### Data analyses

The PET/CT images were reconstructed using the ordered subset expectation maximisation method, with and without attenuation correction. The visual analysis of PET/CT characteristics and pattern was interpreted by at least 2 experienced nuclear medicine physicians (> 20 years of experience in nuclear medicine) who had the patients’ clinical information available on a dedicated workstation. When disputes regarding visual interpretation occurred, consensus was reached by discussion. The ^18^F-FDG PET images were analysed visually and semiquantitatively by calculating the maximal standard uptake value (SUV_max_). The intensity of ^18^F-FDG uptake by cartilages relative to the background and incompatibility with normal anatomy/physiology was assessed, and the intensity was scored using a 4-point visual scale [24, 25]: 0 = absence, not visible on the image display; 1 = faint or less intense than mediastinal blood pool activity; 2 = moderate or equal in intensity to mediastinal blood pool activity; and 3 = more intense than mediastinal blood pool activity. A region of interest (ROI) was placed over the entire area of any abnormal uptake (scales 2 or 3) site. The SUV_max_ was calculated (maximum ROI activity/injected dose per kilogram body weight), and the highest SUV_max_ was used to evaluate the multiple abnormal uptake sites observed in a given lesion or tissue. As the blood glucose level may affect SUV_max_ results, the SUV_max_ needed to be corrected for blood glucose level (SUV_glu_) as follows: SUV_glu_ = SUV_max_ × blood glucose level/130 when the blood glucose level was > 130 mg/dL (7.2 mmol/L) [26]. A positive PET/CT result was indicated by visual score (VS) ≥ 1 or SUV_max_ ≥ 2.0.

### Statistical analysis

Continuous variables were presented as median and range, and qualitative variables as numbers and percentages. Intergroup differences were statistically analysed using SPSS® 17.0 (SPSS Inc., Chicago, IL, USA). Differences in continuous variables between groups were compared using Student’s *t* test. McNemar’s test was used to determine the difference between ^18^F-FDG PET/CT and cartilage biopsy in RP diagnosis. Significance in the statistical analyses was set at a *P* value < 0.05.

## Results

### Patient baseline characteristics

A total of 30 patients (17 men, 13 women) with a median age of 44 years (range, 25–66) were included (range, 25–66 years). The main symptom was cough, which was noted in 24 patients (80%); other symptoms included shortness of breath (15/30, 50%), wheezing (6/30, 20%), excessive sputum (6/30, 20%), fever (4/30, 13.3%), hoarseness (3/30, 10%), chest pain (3/30, 10%), and pharyngalgia (1/30, 3.3%). The duration of these symptoms ranged from 6 months to > 10 years. The detailed baseline characteristics are shown in Table 1.

**Table 1:** Demographic characteristics of the 30 patients with RP

No.	Age (y)/sex	Chief complaint^a^ (duration; months)	Misdiagnosis	Serum levels			ANAs	Lung function			Chest CT scan^b^	Biopsy site
				ESR (mm/1 h)	CRP (mg/dL)	RF (IU/mL)		FEV_1_ (L)	FVC (L)	FEV_1_/FVC (%)		
1	37/M	1 (6)	–	100	22.0	2.1	N	1.68	4.61	36.4	a, b	Tracheal (−)/nasal cartilage (+)
2	38/M	1, 2 (18)	Asthma	115	10.69	3.6	N	1.74	4.69	37.1	a, b	Tracheal (−)/auricular (+)
3	55/M	1, 3 (6)	–	10	0.03	2.4	N	2.57	3.32	77.3	c	Tracheal (−)/nasal cartilage (+)
4	66/F	1, 4 (24)	COPD	105	5.36	4.2	N	1.64	4.56	36.0	e	Tracheal (−)/auricular (+)
5	41/M	1, 4 (9)	–	120	26.41	2.0	N	0.92	1.32	69.7	a, b	Tracheal (−)/auricular (+)
6	55/M	1 (10)	–	112	23.41	2.8	N	1.32	4.19	31.46	a, b	Tracheal (−)/auricular (+)
7	64/M	5 (120)	COPD	11	0.88	4.9	N	0.57	2.37	31.7	a, b	Tracheal (−)/auricular (+)
8	60/M	4 (72)	Amyloidosis	23	1.02	3.4	N	0.49	3.37	92.3	a, b, c	Auricular (+)
9	42/M	1, 4, 6 (4)	–	87	16.2	2.5	N	1.43	4.08	35.0	a, b, c	Tracheal (−)/auricular (+)
10	47/F	1, 4, 7 (1)	–	45	21.0	3.1	N	2.30	4.02	46.5	b	Tracheal (−)/auricular (−)
11	50/F	1 (4); 4 (0.5)	–	34	6.4	3.0	N	1.10	3.67	30.0	b	Tracheal (−)/auricular (+)
12	44/F	1 (4); 4 (1)	Asthma	45	3.4	2.6	N	2.30	4.32	53.2	a, b	Tracheal (−)/auricular (+)
13	44/F	1, 5 (10)	Asthma	68	34.4	3.2	N	1.70	3.89	43.7	a, b	Tracheal (−)/auricular (+)
14	48/F	4 (132)	Asthma	34	10.0	1.0	N	0.67	2.33	28.8	a, b, c	Nasal cartilage (+)
15	25/M	1, 7 (1); 3 (0.5)	–	121	23.0	1.7	N	2.33	4.87	47.8	a, b	Tracheal (−)/auricular (+)
16	26/F	1, 4 (6)	Asthma	56	21.0	2.6	50 U/ml	1.02	3.24	31.5	a, b	Auricular (+)
17	52/M	1, 5, 7 (60)	Asthma	45	19.3	2.5	N	1.05	3.45	30.4	a, b	Auricular (+)
18	43/F	4 (6); 1, 5 (2)	–	34	12.0	1.3	N	2.00	4.01	49.9	a	Nasal cartilage (+)
19	36/F	1, 8 (12); 4, 7 (6)	–	36	11.7	2.6	48 U/ml	1.87	3.56	52.5	a, b	Tracheal (−)/auricular (+)
20	36/M	6 (12); 4 (6)	–	110	34.12	2.1	N	1.56	3.67	42.5	a, b, c	Tracheal (+)
21	42/M	1, 7 (60)	Asthma	34	12.8	1.8	N	2.30	4.50	51.1	a, b	Tracheal (−)/auricular (+)
22	45/M	1, 3 (7)	–	22	11.33	2.3	N	1.20	2.05	58.5	a, d	Auricular (+)
23	34/F	2, 4 (8)	–	56	23.0	3.5	N	1.98	2.87	69.0	a, b, d	Tracheal (−)
24	62/M	1, 7 (36)	COPD	45	21.02	2.5	N	1.56	3.45	45.2	a, b	Tracheal (−)/nasal cartilage (+)
25	45/F	1, 5 (17)	–	33	12.34	1.56	N	2.01	4.56	44.1	a	Tracheal (+)
26	34/F	2, 4 (9)	–	98	23.56	2.01	N	1.87	3.45	54.2	a, b	Tracheal (−)/auricular (+)
27	29/M	1, 3 (8)	–	121	26.71	2.76	N	1.23	3.56	34.6	a, c	Auricular (−)
28	45/M	1, 5 (14)	–	56	11.0	2.04	N	1.34	3.21	41.7	a, b, c	Tracheal (−)/auricular (+)
29	64/M	1, 4 (15)	Asthma	45	32.1	2.87	N	2.23	4.67	47.8	a, b	Tracheal (−)/auricular (+)
30	35/F	1, 6 (12)	–	36	23.1	3.45	N	0.97	1.89	51.3	a, b	Tracheal (−)/auricular (+)

*Chief complaint:* 1 = cough, 2 = chest pain, 3 = fever, 4 = shortness of the breath, 5 = wheezing, 6 = hoarseness, 7 = excessive sputum, 8 = pharyngalgia. *Chest CT scan*: a = diffuse thickening and narrowing of trachea, b = diffuse thickening and narrowing of main bronchus, c = calcification of the trachea, d = enlargement of the mediastinal lymph node, e = no abnormal manifestations

*ANAs* antinuclear antibodies, *COPD* chronic obstructive pulmonary disease, *CRP* C-reactive protein, *ESR* erythrocyte sedimentation rate, *F* female, *FEV*_*1*_ forced expiratory volume in 1 s, *FVC* forced vital capacity, *M* male, *N* none, *P* present, *RF* rheumatoid factor, *RP* relapsing polychondritis, *y* years

### Laboratory test and CT

Erythrocyte sedimentation rate (ESR) and C-reactive protein (CRP) level varied from normal to markedly increased, with a median level of 45 mm/h (range, 10–121 mm/h) and 17.75 mg/dL (range, 0.03–34.40 mg/dL), respectively. ESR and CRP level were increased in some patients and substantially decreased after regular corticosteroid treatment with a median level of 40 mm/h (range, 11–81 mm/h) and 9.78 mg/dL (range, 0.04–20.30 mg/dL), respectively. Antinuclear antibodies were detectable in 2 patients (case 16 and 19) of 50 U/ml and 48 U/ml, but rheumatoid factor was undetectable. The median fasting blood glucose levels before the initial and follow-up scans were 98.5 mg/dL (range, 78–123 mmol/L) and 103 mg/dL (range, 89–121 mmol/L), respectively (*p* = 0.126). Chest CT before diagnosis showed diffuse thickening and narrowing of the trachea in 26 patients (86.7%), narrowing of the main bronchus in 24 (80%), airway wall calcification in 7 (23.3%), and enlargement of the mediastinal lymph node in 2 (6.7%). One patient (3.3%) had no abnormal manifestations (Table 1). Lung function measurements indicated that 25 patients (75%) had obstructive ventilatory dysfunction and 5 (25%) had restrictive ventilation dysfunction.

### ^18^F-FDG PET/CT findings in monitoring disease activity and treatment response

**Fig. 1:**
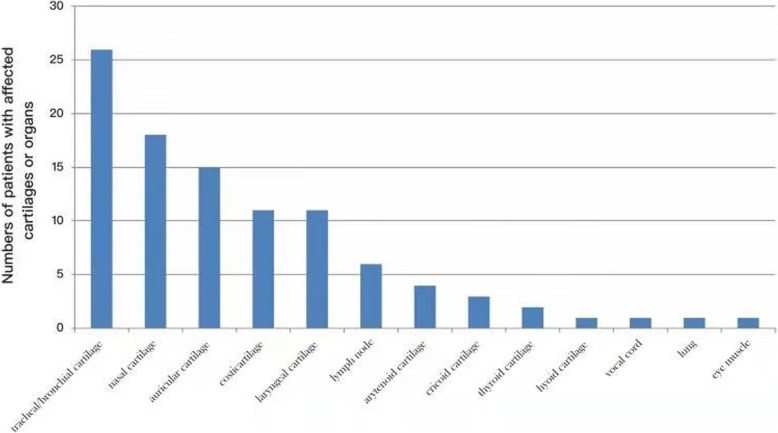
Involved cartilages or organs in 30 patients with RP (in descending order)

The initial ^18^F-FDG PET/CT scan was performed in all patients. Twenty-five patients (83.3%) had increased uptake in > 1 cartilage, and 20 (66.6%) showed increase in ≥ 3 cartilages. The most commonly involved cartilages were tracheal/bronchial (26/30; 86.7%), nasal (18/30; 60%); and auricular cartilages (15/30; 50%). The median VS in the cartilages was 3. SUV_max_ was increased in all patients, except 1 patient, and the median value was 3.8 (range, 1.9–17.9). Figure 1 shows the involved cartilages or organs. When these patients were classified into 2 groups according to the history of corticosteroid treatment, 18 patients without history of therapy were found to have FDG accumulation in ≥ 2 cartilages. However, fewer cartilages were involved than those in patients treated with corticosteroids. Of these 12 patients, 4 (cases 4, 13, 21, and 29) had only 1 cartilage site with intense FDG uptake, and 1 patient (case 7) did not have FDG uptake in any cartilage (Table 2).

**Table 2:** FDG-PET/CT findings of RP in the 30 patients

No.	Initial PET/CT scan					Follow-up PET/CT scan^a^		
	FBG (mg/dL)	Uptake sites^b^	Visual scores	SUV_max_^c^	FBG (mg/dL)	Visual scores	SUV_max_^c^	Interval (months)
1	101	1/2//3/4	3/3//3/3	5.8/5.7/3.6/5.1	116	3/3/1/3	3.1/2.7/−/2.7	3
2	89	1/2/3/4/5/6/7	3/3/3/3/3/3/3	3.8/3.7/4.0/3.9/4.0/3.8/3.7	101	2/2/2/0/3/0/0	2.0/2.1/2.1/−/3.1/−/−	3
3	83	2/3/4/6/7/8	3/3/3/3/3/3	4.4/4.2/3.8/3.8/3.8/4.8	98	3/0/3/3/3/3	3.8/−/3.0/3.0/3.0/3.0	2.5
4	93	6	3	3.6				
5	93	1/3/8/9	3/3/3/3	3.3/4.2/4.5/5.0	89	2/0/3/3	2.1/−/3.0/2.8	3
6	115	1/8	3/3	3.5/4.2	108	3/0	2.8/−	4
7	85	–	–	–				
8	116	1/9	3/3	3.9/2.5				
9	88	1/9/10	3/3/3	3.3/4.5/4.3				
10	79	1/10	3/3	3.1/4.3				
11	112	1/8	3/3	3.3/3.8				
12	101	1/3/8/11	3/3/3/3	5.9/2.8/3.2/5.7	121	3/0/0/2	2.8/−/−/2.0	3
13	122	10	3	4.0				
14	99	1/2	3/3	3.4/3.8				
15	87	1/2/8/10	3/3/3/3	3.8/4.9/4.9/3.5				
16	110	1/2/3/9/10	3/3/3/3/3	2.5/2.6/2.2/4.7/2.6				
17	95	1/2/8/12	1/3/3/3	−/6.2/3.2/2.7	113	0/2/0/0	−/3.0/−/−	4
18	123	1/2/8/10	1/2/3/3	−/1.9/3.0/2.2				
19	101	1/2/8/10	3/3/3/3	2.7/3.7/3.0/5.5				
20	111	1/2/4/8/10	3/3/3/3/3	3.2/3.2/4.2/4.0/4.2	110	0/3/0/2/0	−/3.9/−/2.0/−	3
21	121	1	3	2.0				
22	96	1/2/9	3/1/3	3.8/−/6.4				
23	88	1/2/3/8/9/10	3/3/3/3/3/3	3.7/3.0/4.5/3.5/3.5/3.2				
24	99	1/2/3/8/10	3/3/3/3/3	3.8/2.7/8.1/3.6/7.2	103	0/0/0/3/0	−/−/−/4.2/−	4
25	78	1/2/3/8	3/3/3/3/3	2.2/3.3/4.0/4.3				
26	108	1/2/3/8/10	3/2/3/2/3	4.8/1.8/2.8/1.7/3.8				
27	97	1/2/8/10	3/3/3/3	4.0/2.9/3.9/4.9	102	0/0/1/3	−/−/1.2/2.8	3
28	87	1/2/13	3/3/3	9.1/4.9/17.9				
29	98	1	2	2.0				
30	102	1/2/3/9/10	3/3/3/3/3	4.6/3.2/4.5/4.7/5.0				

*FDG-PET/CT* fluorodeoxyglucose positron emission tomography/computed tomography, *FBG* fasting blood glucose, *SUV*_*max*_ maximum standardised uptake value

^a^Results of the second PET/CT scan, even though 3 follow-up scans could be performed

^b^*Uptake sites*: 1 = tracheal/bronchial cartilage; 2 = nasal cartilage; 3 = costicartilage; 4 = arytenoid cartilage; 5 = hyoid cartilage; 6 = cricoid cartilage; 7 = thyroid cartilage; 8 = auricular cartilage; 9 = lymph node; 10 = auricular cartilage; 11 = vocal cord; 12 = lung; 13 = eye muscle

^c^If the FDG uptakes were in different positions of the same cartilage, SUV_max_ should be representative of the highest one

**Fig. 2:**
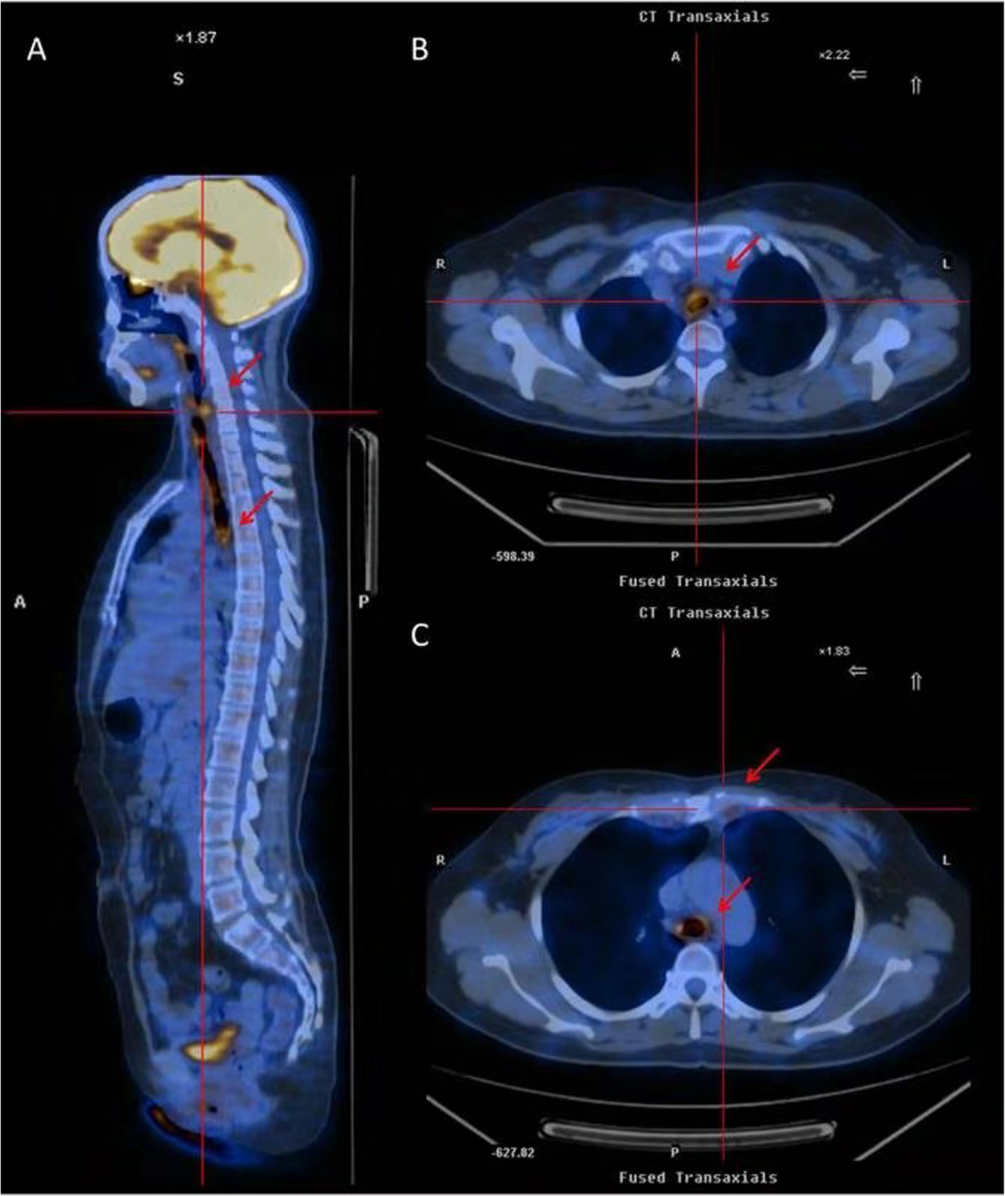
A 44-year-old woman with RP showed multicartilage involvement. **a** A whole-body view showed multiple foci of pathological tracer uptake in cartilages (vocal cord, SUV_max_ 5.8; tracheal/bronchial cartilage, SUV_max_ 5.8; red arrowhead). **b** The SUV_max_ of the tracheal cartilage was approximately 5.8 (red arrowhead). **c** Abnormal uptake was detected in the costicartilage and tracheal cartilage (red arrowhead)

Ten patients underwent second PET-CT scans after 2.5–15 months of steroid-based therapy. Most symptoms, such as cough, shortness of breath, excessive sputum, and fever, were relieved. After systemic corticosteroid therapy, 8 patients had complete therapeutic response, while 2 patients had partial response. One patient had relapse and died in the 3-year follow-up due to tracheal stenosis. Most importantly, VS (2 vs 3, *p* < 0.0001) and SUV_max_ (2.9 vs 3.8, *p* < 0.001) significantly decreased. FDG uptake decreased or disappeared during corticosteroid therapy in a majority of patients. FDG uptake diminished in most involved cartilages, except in the auricular cartilage of case 24 but with complete therapeutic response (Fig. 2).

### Cartilage biopsy findings

**Fig. 3:**
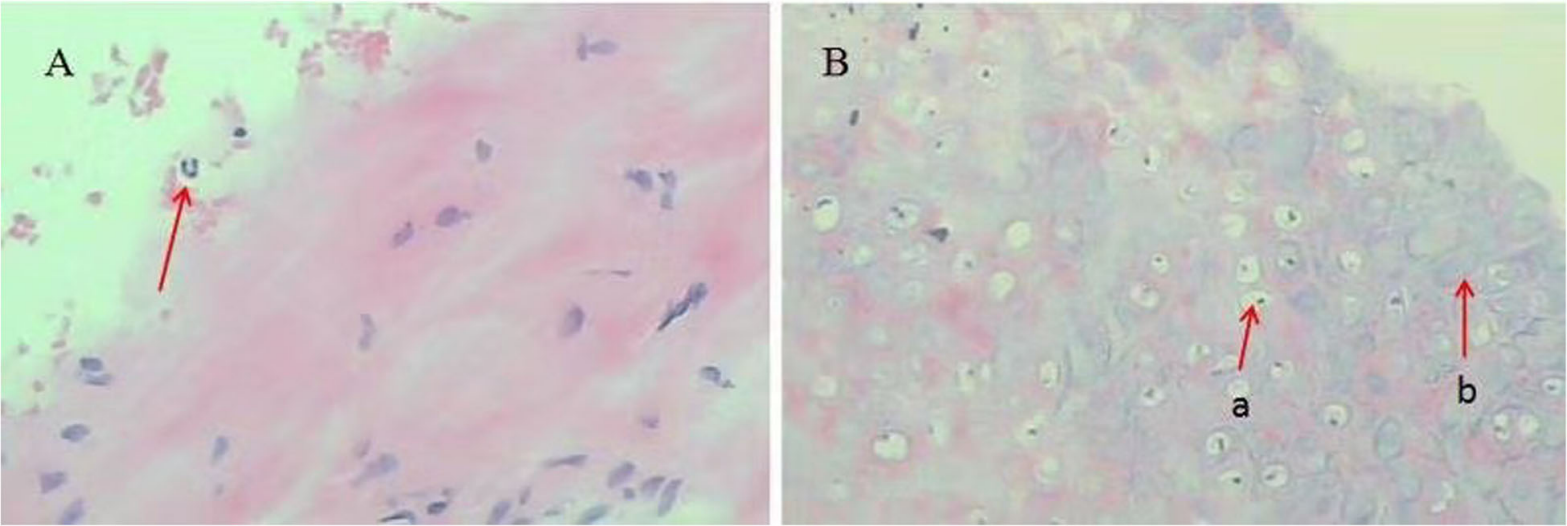
A biopsy sample obtained from the auricular cartilage showed characteristic RP pathology. **a** H&E staining of neutrophils (red arrowhead). **b** H&E staining of normal (**a**) and necrotic chondrocytes (**b**)

The results of cartilage biopsies performed at different sites in all patients are shown in Table 3. ^18^F-FDG PET/CT showed 5 true positives for nasal cartilage in 5 patients. Bronchoscopy with the aid of PET/CT was performed in 19 patients. However, based on biopsy-proven sites (tracheal/bronchial cartilage), the sensitivity of PET/CT was 10.53% (2/19). Four patients who underwent biopsies of the tracheal/bronchial cartilage by bronchoscopy all had negative findings (0/4, 0%). Eight (88.9%) of 9 biopsy-proven sites (auricular cartilage) were detected using ^18^F-FDG PET/CT. Figure 3 presents the histology of a patient. Even without the assistance of PET/CT, the positive rate of auricular cartilage biopsy was up to 92.31% (12/13). Therefore, no correlation was found between PET/CT and biopsy in RP diagnosis with respect to the auricular cartilage.

**Table 3:** Correlation of PET/CT with cartilage biopsy in diagnosing RP

PET/CT^a^	Cartilage biopsy		Total
	Positive	Negative	
Nasal cartilage			
Positive	5	0	5
Negative	0	0	0
Total	5	0	5
McNemar’s chi-square test: *χ*^2^ = NaN; *p* = NaN			
Auricular cartilage			
Positive	8	1	9
Negative	12	1	13
Total	20	2	22
McNemar’s chi-square test: *χ*^2^ = 7.69; *p* = 0.005			
Tracheal/bronchial cartilage			
Positive	2	17	19
Negative	0	4	4
Total	2	21	23
McNemar’s chi-square test: *χ*^2^ = 15.1; *p* = 0.001			

*PET/CT* positron emission tomography/computed tomography, *NaN* not a number, *RP* relapsing polychondritis

^a^PET/CT-guided biopsy was performed in affected cartilage when PET/CT showed increased FDG uptake

## Discussion

RP is a rare inflammatory chondropathy in multiple cartilages that is not readily diagnosed in the absence of typical clinical findings [2, 20, 27–31]. The diagnosis of RP is still based on clinical grounds [4, 5], and histologic confirmation is not required. According to Damiani and Levine [6], the diagnosis can be established when one or more clinical features are present in conjunction with biopsy confirmation. Our previous study revealed that PET/CT is a powerful tool in diagnosing RP as it can detect multisystemic cartilaginous abnormalities via increased FDG uptake [20]. Yamashita et al. [21] found several specific patterns of PET/CT findings in RP, such as FDG uptake in bilateral auricular cartilage, and FDG uptake was noted in various combinations of affected lesions in a single patient. These authors believe that the specific PET/CT findings may be of high diagnostic value. However, the number of patients in these studies was small. To our best knowledge, the current study has the largest patient population. Significantly, PET/CT showed that 83.3% (25/30) of patients had disease involvement in > 1 cartilage. We also found that all 18 patients without history of corticosteroid treatment had involvement in > 1 cartilage. Therefore, FDG uptake in multiple cartilages, such as tracheal/bronchial, nasal and auricular cartilages, in a single patient suggests the diagnosis of RP, and a biopsy might not be necessary. However, the other 12 patients who received irregular corticosteroid treatment previously had fewer involved cartilages detected by PET/CT, and 4 of them had only one site of intense FDG uptake in cartilages. One patient who had received corticosteroid treatment did not exhibit intense FDG uptake in any cartilages. Thus, repeated administration of corticosteroids may undermine the diagnostic value of ^18^F-FDG PET/CT.

Although biopsy of inflamed sites is not routinely performed, the detection of basophilic staining of the cartilage matrix, perichondral round-cell infiltration, or cartilage destruction with fibrous replacement can have diagnostic value [32]. Based on the criteria by Damiani and Levine, who expanded the spectrum diagnostic criteria, positive histologic confirmation is helpful in RP diagnosis. In our respiratory centre, definitive diagnosis of RP is usually made according to both clinical manifestation and biopsy findings, especially in patients with atypical clinical presentation or in early stages. The main biopsy sites are the auricular and nasal cartilages, which are relatively easy and safe to obtain. We found that the positive rate of PET/CT-guided biopsy was 100% in the nasal cartilage (5/5) and 88.9% in auricular cartilage (8/9), and this rate was 92.3% in the auricular cartilage even without PET/CT (12/13). This finding reveals that PET/CT is not sensitive and specific in the identification of patients with RP with auricular involvement. A possible explanation is that FDG accumulates less in the auricular cartilage due to poor blood supply.

In terms of examining tracheal involvement, PET/CT is sufficient to provide accurate visualisation of RP by demonstrating increased FDG uptake in tracheal cartilages, airway wall thickening, airway stenosis, airway malacia, airway wall calcification, and air trapping. In our study, the positive rate of PET/CT-guided biopsy was low in the tracheal/bronchial cartilage (10.5%), even though FDG uptake was high. Therefore, a positive PET/CT finding is inconsistent with biopsy finding in the tracheal/bronchial walls by bronchoscopy. The reason for such difference may lie in the technical difficulty in accessing tracheal/bronchial cartilages when there is tracheal collapse, fibrosis, or stenosis. Bronchoscopy is invasive and may consequently exacerbate mucosal swelling or cartilage inflammation via mechanical stimulation [7]. Serious complications arising from cartilage biopsies of the trachea or bronchus in patients with RP have been reported [33]. Therefore, the value of PET/CT-guided biopsy seems limited in inaccessible sites that are of clinical significance.

Corticosteroid therapy is the mainstay of treatment for RP, and some researchers have reported that PET/CT is valuable for monitoring the treatment response. Lei et al. [22] noted that post-treatment PET/CT showed obvious decreases or complete disappearance of high ^18^F-FDG uptake lesions in 10 of 22 cases, which were highly consistent with symptom improvement, and Yamashita et al. [21] reported similar findings. In our study, a follow-up scan performed in 10 (33%) of 30 patients showed that median VS and SUV_max_ were significantly decreased after therapy. These changes were also correlated with clinical symptom improvement and laboratory tests.

These findings suggest that PET/CT is a promising radiological tool in monitoring treatment response and disease progression. However, because there is no agreement regarding the duration of corticosteroid treatment, there is no consensus regarding the use of PET/CT to optimise corticosteroid strategies, although some studies have revealed its potential value [10–16]. The interval for PET/CT scans after corticosteroid therapy remains unclear, as it has been reported to range from 1 month to 13 months [10–12, 14, 21]. To our best knowledge, there is no evidence that PET/CT can provide prognostic information in patients with RP and could be considered a guide for making treatment decisions. In 2 of our patients, in whom follow-up data indicated symptomatic and inflammatory improvement, FDG uptake diminished in most affected sites but increased in 2 other sites (nasal and auricular cartilages). Thus, in some patients, PET/CT results may differ depending on their symptoms. In some cartilage sites, FDG accumulation after corticosteroid treatment of patients with RP may be correlated with other factors (except inflammatory activity). In the present study, only 33.3% of patients (10/30) underwent follow-up PET/CT, mainly because of radiation exposure and high cost in developing countries, and further studies involving larger numbers of patients are necessary to evaluate the role of follow-up PET/CT scans during treatment. Thus, the cost-effectiveness of repeated PET/CT scans and the resultant radiation exposure must also be considered.

Our study has some limitations. First, it is a retrospective study; therefore, the results may not completely translate to clinical practice. Second, because all patients included in the study presented with respiratory symptoms, it is possible that we underestimated the value of PET/CT in patients with RP without respiratory involvement. Lastly, the number of patients was still extremely small to reflect the low incidence of this disease and well-defined patient populations. Thus, a larger, multicentre, prospective, randomised study is needed for further validation of our results.

## Conclusions

^18^F-FDG PET/CT helps identify multiple cartilage involvement in RP but seems neither sensitive nor specific in evaluating RP with airway involvement. Moreover, PET/CT has limited utility in locating biopsy sites and monitoring response to corticosteroid treatment.

## Data Availability

The datasets used and/or analysed during the current study are available from the corresponding author on reasonable request.

## Abbreviations

^18^F-FDG PET/CT: ^18^F-fluorodeoxyglucose positron emission tomography/computed tomography
ANA: Antinuclear antibody
COPD: Chronic obstructive pulmonary disease
CRP: C-reactive protein
ESR: Erythrocyte sedimentation rate
HRCT: High-resolution computed tomography
OSEM: Ordered subset expectation maximisation method
RF: Rheumatoid factor
ROI: Region of interest
RP: Relapsing polychondritis
SUVmax: Maximal standard uptake value
TBNA: Transbronchial needle aspiration
VS: Visual score
